# Sociodemographic Determinants of Nonadherence to Depression and Anxiety Medication among Individuals Experiencing Homelessness

**DOI:** 10.3390/ijerph18157958

**Published:** 2021-07-28

**Authors:** Sahar S. Eshtehardi, Ashley A. Taylor, Tzuan A. Chen, Marcel A. de Dios, Virmarie Correa-Fernández, Darla E. Kendzor, Michael S. Businelle, Lorraine R. Reitzel

**Affiliations:** 1 Department of Psychological, Health, & Learning Sciences, The University of Houston, 3657 Cullen Blvd Stephen Power Farish Hall, Houston, TX 77204, USA; sseshteh@central.uh.edu (S.S.E.); aataylo4@central.uh.edu (A.A.T.); tchen38@uh.edu (T.A.C.); madedios@uh.edu (M.A.d.D.); vcorreafernandez@uh.edu (V.C.-F.); 2 HEALTH Research Institute, The University of Houston, 4849 Calhoun Rd., Houston, TX 77204, USA; 3 TSET Health Promotion Research Center, The University of Oklahoma Health Sciences Center, 655 Research Parkway, Suite 400, Oklahoma, OK 73104, USA; darla-kendzor@ouhsc.edu (D.E.K.); michael-businelle@ouhsc.edu (M.S.B.)

**Keywords:** medication adherence, homeless, depression, anxiety, health disparities

## Abstract

Psychiatric medication nonadherence continues to be a leading cause of poor health outcomes for individuals experiencing homelessness. Identifying the sociodemographic factors that contribute to medication nonadherence may help guide strategies to care for and support this group. This study examined 200 adults with depression diagnoses and active anti-depressant prescriptions (Mage = 43.98 ± 12.08, 59.4% Caucasian, 58.5% male, 70% uninsured, 89.5% unemployed) and 181 adults with anxiety diagnoses and active anti-anxiety prescriptions (Mage = 43.45 ± 11.02, 54.4% Caucasian, 57.5% male, 66.3% uninsured, 88.9% unemployed) recruited from six homeless-serving agencies in Oklahoma City. Self-reported sociodemographic variables included: age, sex, race/ethnicity, education, monthly income, employment status, and health insurance status. Adjusted logistic regression analyses revealed that employed (OR = 4.022, CI_0.95_: 1.244–13.004) and insured (OR = 2.923, CI_0.95_: 1.225–6.973) participants had greater odds of depression medication nonadherence. For anxiety, being employed (OR = 3.573, CI_0.95_: 1.160–11.010) was associated with greater odds of anxiety medication nonadherence, whereas having depression and anxiety diagnostic comorbidity (OR = 0.333, CI_0.95_: 0.137–0.810) was associated with lower odds of anxiety medication nonadherence. Interventions aimed at facilitating accessible prescription acquisition or otherwise reducing barriers to prescription medications for employed adults, including those with health insurance, may benefit adherence, but more research is needed. Future studies would benefit from using a qualitative approach to better delineate nuanced barriers to psychiatric medication adherence.

## 1. Introduction

Medication nonadherence, or the extent to which an individual’s health behaviors correspond with agreed pharmacological treatment recommendations from a health care provider, continues to be a leading cause of poor health outcomes across the United States [1]. Current research argues that optimizing adherence to medical interventions may have a greater impact on overall health outcomes when compared to any specific medical treatment [2]. However, nearly 50% of chronically ill patients do not adhere to the medication regimen recommended by their healthcare providers, with nonadherence rates typically higher in patients with acute conditions [3]. Furthermore, multifaceted treatment plans involving multiple daily doses, possibly with varying dosage levels, require the patient to modify their behaviors, which is associated with even greater likelihood of nonadherence (i.e., 70%) [4]. Medication nonadherence also carries a massive economic burden [4]. Estimated in the hundreds of billions of dollars, the fiscal ramifications of medication nonadherence include high costs to health care systems, including preventable emergency department visits and hospitalizations [1]. Hospitalizations due to medication nonadherence, for example, have been estimated to cost approximately 13.35 billion USD annually in the United States [4]. The Centers for Medicare and Medicaid Services have adopted strategies to combat medication nonadherence, integrating these strategies into its Star Ratings program and stand-alone prescription drug plans [2]. Despite literature supporting the importance of medication adherence for optimal health outcomes and economic expenditures, improving medication adherence continues to be a challenge across disease groups [5], medication types [6], and healthcare settings [7].

### 1.1. Medication Nonadherence among Individuals Experiencing Homelessness

Marginalized individuals, including homeless and vulnerably housed adults, are at an increased risk of sub-optimal health outcomes as a result of medication nonadherence. Homeless individuals face several unique barriers to medication adherence including financial instability, limited or lack of insurance coverage, lack of storage space for medications, and issues around transportation to acquire prescriptions [8]. Previous literature has explored medication nonadherence among individuals experiencing homelessness focusing on populations taking medications for human immunodeficiency virus (HIV) [9,10,11,12], schizophrenia [13,14,15], and bipolar disorder [16]. Rates of nonadherence range from 22% to 49% for these medications, each of which may have significant side effects, further contributing to nonadherence in this population over and above the practical challenges previously described. Case in point, Kidder and colleagues [11] assessed antiretroviral medication adherence rates among 7925 homeless and vulnerably housed individuals across 19 urban United States sites and found that individuals experiencing homelessness reported higher nonadherence rates over a 30-day-period when compared to domiciled individuals (44.3% vs. 31.9%). Medication nonadherence among this vulnerable group may be a significant contributor to their well-established burden of premature morbidity and mortality [17].

Adherence to psychiatric medication is also critical for favorable health outcomes [18]. Of the estimated 553,000 homeless individuals currently in the United States, 24% suffer from serious mental illnesses that require daily medication management [19]. Failure to adhere to psychiatric treatment regimens often leads to worsening of the disease and poor quality of life [7]. Nonadherence to psychiatric medications, such as anti-depressants and anti-anxiety medications can lend itself to increased risk of disease progression, suicide attempts, and hospitalizations contributing to premature morbidity and mortality [15,20,21,22]. However, little research has focused on psychiatric medication nonadherence, especially medications prescribed for depression and anxiety, among individuals experiencing homelessness. Furthermore, estimates from prior studies suggest that depression and anxiety comorbidity is common (affecting > 50% of individuals who are homeless), supporting the need for a better understanding of the factors associated with nonadherence to these medications [8].

### 1.2. Sociodemographic Factors and Medication Nonadherence among Individuals Experiencing Homelessness

Previous studies of medication nonadherence across populations have long focused on individual characteristics including age, sex, race/ethnicity, education level, income, employment status, and insurance status to tailor interventions aimed at increasing adherence rates and improving health outcomes [2,3,7]. Despite this, to date, only a few studies within homeless samples have examined the role of sociodemographic factors on medication nonadherence, with even fewer studies examining nonadherence rates for psychiatric medication within this vulnerable population [1,8,14]. For example, Hunter and colleagues examined nonadherence across a range of prescription medications including psychiatric medications, grouped together, among sheltered homeless and vulnerably housed adults within three Canadian cities [1]. They reported that being male, 40 years of age or less, employed, and of lower educational achievement were associated with higher rates of nonadherence. In contrast to reported findings with domiciled samples [23], income had no effect on adherence rates in this homeless/vulnerably housed sample [1]. Although this study enhanced understanding of the sociodemographic factors affecting medication nonadherence among individuals who were homeless, it is important to consider that data from other countries may not be generalizable to the United States based on differences in prescription medication coverage, availability, access, etc. Research from Coe et al. [8] examined psychiatric medication nonadherence among homeless adults in Virginia, USA, and found that individuals aged 49 or younger reported higher rates of nonadherence when compared to adults 50 years of age and older. In contrast to prior research with homeless/vulnerably housed adults [1], however, findings from this study suggested no sex differences in psychiatric medication nonadherence. Moreover, work by Coe et al. [8] did not report on education level, income, nor employment status—all of which have been shown within domiciled samples to affect overall medication adherence rates [7]. In another study on the topic, Tinland and colleagues [14] examined schizophrenia medication nonadherence among homeless and vulnerably housed adults in four French cities. Contrary to the previous studies [1,8], authors found that homeless women with a diagnosis of schizophrenia had higher rates of nonadherence when compared to men, primarily due to increased subjective feelings of undesirable side effects. Additionally, medication nonadherence rates in the Tinland and colleagues [14] study increased with age, unemployment, and low education levels, particularly in individuals without a bachelor’s degree.

In summary, the literature shows mixed results regarding the associations of sociodemographic variables with psychiatric medication nonadherence among individuals experiencing homelessness, and few studies on this topic were United States-based. Specifically, sex and employment differences in medication nonadherence rates were a major source of inconsistency within the literature [1,8,14]. Additionally, no prior study amongst individuals experiencing homelessness has examined the role of health insurance status on psychiatric medication nonadherence, despite the distinct role this could play in access to medications. Notably, many of these studies did not report nonadherence by diagnosis, instead grouping all psychiatric diagnoses together to examine factors associated with overall medication nonadherence. Given that medication nonadherence rates differ between psychiatric conditions, however, it may be additionally important to examine nonadherence by psychiatric diagnosis [24]. Furthermore, despite the high prevalence of depression and anxiety disorders among the homeless population and the importance of adherence for optimal treatment outcomes, no prior research has focused specifically on depression and anxiety medication nonadherence. Thus, research delineating the role of sociodemographic factors on psychiatric medication nonadherence rates within a homeless sample would be an instrumental first step toward better understanding, and ultimately addressing, the associated physical and mental health disparities within this marginalized group.

### 1.3. Current Study

To our knowledge, no other study has specifically examined the sociodemographic factors related to depression and anxiety medication nonadherence, respectively, among individuals experiencing homelessness in the United States. The primary purpose of the current study is to address this gap in the literature. The secondary purpose of the present study is to identify barriers to depression and anxiety medication adherence unique to individuals experiencing homelessness. Thus, the aims and hypotheses of the current study are as follows:

**Aim** **1.** *Examine the association between sociodemographic characteristics (age, sex, race/ethnicity, education, income, employment status, and health insurance status) and depression and anxiety medication nonadherence in a subset of a diverse sample of homeless adults who were under active psychotropic prescription.*

**Hypothesis** **1.** *We hypothesized that younger age, male sex, minority status, less education, unemployment, and lower income would be associated with greater odds of nonadherence, regardless of diagnosis. Due to the lack of literature and/or prior mixed findings on income, we did not purport directional hypotheses for this variable.*

**Aim** **2.** *Describe self-reported barriers to depression and anxiety medication adherence in this sample of adults experiencing homelessness.*

**Hypothesis** **2.** *In line with previous research conducted among homeless individuals [1], we hypothesized that the most commonly endorsed barriers to depression and anxiety medication adherence would include an inability to fill prescriptions and to pick up the prepared prescription from the pharmacy.*

## 2. Materials and Methods

### 2.1. Participants

Participants were recruited via study flyers posted at six agencies providing services and/or shelter to homeless individuals in Oklahoma City, Oklahoma, USA. Flyers advertised the need for volunteers for a study focused broadly on health factors associated with experiencing homelessness. The following were inclusion criteria: (a) minimum age of 18 years, (b) currently receiving services (e.g., food, shelter, counseling) at the targeted shelters/agencies, and (c) at least a seventh-grade English literacy level as indicated by a score of ≥4 on the Rapid Estimate of Adult Literacy in Medicine-Short Form [25].

### 2.2. Procedures

Screening for eligibility took place at each study site on specified data collection days. Of the 648 screened individuals, 38 were deemed ineligible due to an insufficient literacy level. Eligible individuals (*n* = 610) provided informed consent and completed questionnaires via a tablet computer whereby survey items were visible on the screen and read aloud over headphones using Questionnaire Development System software, version 3.0 (Nova Research, Silver Springs, MD, USA). Study staff were available to assist in the event of technical problems, but none were reported. Data collection took place at the recruitment sites and occurred in July and August of 2016. Participants were compensated with a $20 department store gift card for their participation. The current analyses further excluded 29 participants because they reported that they were not currently homeless, resulting in the total potential sample size of 581.

### 2.3. Measures

#### 2.3.1. Sociodemographic Characteristics

The following variables were collected via self-report: age, sex, race/ethnicity, years of education, last month’s income, employment status, and current health insurance status.

#### 2.3.2. Medication Adherence

Items to assess psychiatric medication nonadherence were investigator-developed self-report questions. Items assessed historical and/or current psychiatric diagnosis and current active psychiatric prescription status. Each of the above were asked with regard to depression and anxiety disorders (excluding PTSD), respectively. Specifically, nonadherence to depression medication and anxiety medication were each assessed with a single item asking participants whether they took their prescribed medication the previous day. This item read: “*Did you take your [insert psychiatric illness] medication yesterday?*” (no versus yes). Additionally, we created a prescription comorbidity item (taking either depression or anxiety medication prescription versus taking both depression and anxiety medication prescriptions).

#### 2.3.3. Barriers to Adherence

If participants reported psychiatric medication nonadherence, factors related to nonadherence were queried. This item read: “*What were the reasons you did not take your [insert psychiatric illness] medication yesterday? (check all that apply)*”. Response choices included: side effects; forgot; the medication is not working; I do not need the medication anymore; I have not picked up the medication from the pharmacy; I cannot get the medication refilled; and other reasons.

### 2.4. Analytic Plan

The descriptive statistics of participants’ characteristics and study variables were assessed, and the comparisons between medication adherence statuses were examined using t-test or chi-square test for continuous and categorical variables, respectively. Logistic regressions were used to measure associations between medication adherence status and age, sex, race/ethnicity, education, income, employment status, health insurance status, and depression/anxiety comorbidity. The analyses were conducted on a subset of participants with an active depression or anxiety medication prescription, and the analyses were run separately for depression and anxiety. Simple frequency analyses were run to identify barriers to taking psychiatric medication, as reported by those who reported nonadherence to their psychiatric medication. Correlations and chi-square tests were used to examine associations between participants’ characteristics and barriers to taking psychiatric medications. All analyses were conducted using SAS version 9.4 [26] with a statistical significance level designated at *p* < 0.05.

## 3. Results

Of the 610 total participants in the parent study, 581 were currently homeless. This study examined subsets of homeless participants with an active depression medication prescription (*n* = 200, 34.42%) or anxiety medication prescription (*n* = 181, 31.15%), with some participants overlapped between these groups.

### 3.1. Depression Medication Nonadherence

Of the 200 participants who were prescribed medication for depression, 58.50% (*n* = 117) were men. Overall, 59.39% of this group self-identified as being Non-Hispanic White, 16.24% as Black or African American, 8.63% as Native American/Alaska Native, and 15.74% as Hispanic/multi-racial/other. Additionally, 89.5% (*n* = 179) reported they were unemployed, 70% (*n* = 140) reported they were uninsured, and 72.5% (*n* = 145) had depression/anxiety comorbidity and were prescribed medications to address each condition. Among those prescribed depression medication, 23.5% (*n* = 47) reported nonadherence to those medications over the prior day. Participant characteristics by medication adherence status are shown in Table 1.

Chi-square tests of independence revealed significant associations between depression medication nonadherence and last month’s income (*p* = 0.0342), race/ethnicity (*p* = 0.0018), and insurance status (*p* = 0.0012), respectively. Those reporting depression medication nonadherence were more likely to have greater monthly income (USD 481.98 vs. USD 232.52), health insurance (48.94% vs. 24.18%), and were less likely to be Non-Hispanic White (47.83% vs. 62.91%) or Hispanic/multi-racial/other (6.52% vs. 18.54%) relative to participants who indicated adherence to depression medication.

Logistic regression analyses revealed that participants who were employed and insured had 4.022 and 2.923 times greater odds of depression medication nonadherence. Compared with Non-Hispanic White participants, African American and Native American/Alaska Native participants had greater odds of depression medication nonadherence with adjusted odds ratios of 3.371 and 3.713, respectively (Table 2).

### 3.2. Anxiety Medication Nonadherence

Of the 181 participants who were prescribed medication for anxiety, 57.46% (*n* = 140) were men. Overall, 54.44% (*n* = 98) of this group self-identified as being Non-Hispanic White, 17.22% were Black or African American, 11.11% were Native American/Alaska Native, and 17.22% were Hispanic/multi-racial/other. Additionally, 88.95% (*n* = 161) were unemployed, 66.3% (*n* = 120) were uninsured, and 80.11% (*n* = 145) had depression/anxiety comorbidity and were prescribed medications to address each condition. Among those prescribed anxiety medication, 33.15% (*n* = 60) indicated nonadherence to those medications over the prior day. Participant characteristics by medication adherence status are shown in Table 3.

Chi-square tests of independence revealed significant associations between anxiety medication nonadherence and last month’s income (*p* = 0.0424), race/ethnicity (*p* = 0.0253), insurance status (*p* = 0.0235), and depression/anxiety prescription comorbidity (*p* = 0.0003), respectively. Those reporting anxiety medication nonadherence were more likely to have greater income (USD 513.35 vs. USD 272.88) and health insurance (45.00% vs. 28.10%). Conversely, those reporting anxiety medication nonadherence were less likely to report depression/anxiety prescription comorbidity (65.00% vs. 87.60%) and self-identify as Non-Hispanic White (45.76% vs. 58.68%) or Hispanic/multi-racial/other (11.86% vs. 19.83%) relative to participants who indicated adherence to anxiety medication.

Logistic regression analyses revealed that participants who were employed had greater odds (adjusted OR: 3.573), whereas those with depression/anxiety prescription comorbidity had lower odds (adjusted OR: 0.333) of anxiety medication nonadherence (Table 4).

### 3.3. Barriers to Adherence

The frequency of the reasons for nonadherence to each psychiatric medication are presented in Table 5. The most commonly endorsed reason for depression and anxiety medication nonadherence was “other” (31.91% and 36.67%, respectively), followed by “cannot get the medication refilled” (23.40% and 30.00%, respectively) and “haven’t picked up from pharmacy” (23.40% and 16.67%, respectively).

Results of the correlational and chi-square analyses of participant characteristics and barriers to adherence indicated that: (1) participants with less education were more likely to endorse “forgot” as their reason for not taking depression medication (r = −0.37070, *p* = 0.0103); (2) participants reporting “other reasons” for not taking depression medication were more likely to have insurance (73.33% vs. 26.67%; x^2^ = 5.2478, *p* = 0.0220); and (3) participants with less education were more likely to endorse “medication not working” as their reason for not taking anxiety medication (r = −0.33988, *p* = 0.0079).

## 4. Discussion

The literature supports the notion that medication nonadherence has continued to be a leading cause of poor health outcomes across the United States, particularly among individuals experiencing homelessness [1]. This study was the first, to the authors’ knowledge, to explore the association between sociodemographic characteristics and depression and anxiety medication nonadherence among a large and diverse sample of adults experiencing homelessness. Results indicated that depression medication nonadherence was associated with having health insurance and being employed—even after controlling for other sociodemographic variables and depression/anxiety prescription comorbidity. Specifically, those who were employed and insured were four times and nearly three times more likely to endorse depression medication nonadherence, respectively. Our results were similar to those of Hunter et al. [1], who found that within three Canadian cities, sheltered homeless and vulnerably housed individuals who were employed had higher rates of medication nonadherence. Likewise, anxiety medication nonadherence was associated with employment status in adjusted analyses whereby those who were employed were in excess of three times more likely to endorse anxiety medication nonadherence. The literature suggests that those who are employed tend to experience higher rates of medication nonadherence when compared to those who are unemployed, which may be a result of competing demands on time, an area worthy of further exploration [27].

Overall, results may suggest that interventions aimed at facilitating accessible prescription acquisition or otherwise reducing barriers to prescription medications for employed adults experiencing homelessness, including those with health insurance, may benefit their adherence. This may be especially the case given that some of the more commonly endorsed reasons for nonadherence included not picking up medication from the pharmacy and/or not refilling prescriptions. More research is needed; however, if true, homeless shelters and homeless-serving agencies might adopt “runners” to drop off and pick up prescriptions for their stakeholders as one way to address this issue. Results may also suggest that more assistance is needed returning to the doctor for refills after an initial prescription. Alternatively, our pattern of results might also suggest that employment itself is a barrier to medication adherence—perhaps because medication side effects impact job performance; however, only a small proportion of non-compliant respondents endorsed side effects as the reason for their behaviors. Overall, shelter and homeless-serving agencies should make attempts to ensure that they make prescribed medications as accessible as possible (e.g., stored in a locker that only employees can access) to individuals who may have varied work schedules as a way to potentially facilitate adherence.

Interestingly, having depression/anxiety prescription comorbidity was associated with lower odds of anxiety medication nonadherence among this vulnerable group. This finding may align with prior research conducted in Sweden amongst a domiciled population supporting the notion that being anxious about health can improve medication adherence [28]. Or, perhaps having to take more prescriptions simply leads to greater recall about the need to take medications that translates into greater adherence, but more research on this is needed given that this comorbidity only predicted anxiety medication adherence.

Our findings also highlighted the existence of racial/ethnic disparities related to depression medication nonadherence. Consistent with what was hypothesized, those who self-identified as Black or African American or Native American/Alaska Native were more than three times as likely to report depression medication nonadherence when compared to their Non-Hispanic White counterparts. This pattern, however, was not associated with anxiety medication adherence in adjusted analyses. Additional context may be needed to understand these results.

Broad and inexpensive medication adherence reminders (e.g., a sign at the exit of a shelter reminding guests “did you remember to take your medications today?”) might serve to benefit residents overall and in doing so be of particular value to specific groups of residents at higher risk of nonadherence. “Forgetting” to take medications did not appear to be among the most endorsed barriers to adherence, but it was the fourth and fifth most common reasons for depression and anxiety medication nonadherence, respectively. In any case, signs about medication adherence may also “normalize” psychiatric prescriptions to some degree, in the event that nonadherence may be related to stigma within some racial/ethnic groups [29]. Additionally, individuals with less education endorsed “forgot” as their reason for not taking their depression medication and “medication not working” as their reason for not taking their anxiety medication, respectively. Thus, interventions centered on strategies to combat forgetfulness, as well as psychoeducational interventions aimed at increasing patient knowledge as it pertains to their psychiatric medications and anticipated expectations of medication efficacy. Furthermore, the high endorsement of “other reasons” for depression and anxiety medication nonadherence may or may not relate to the experience of stigma; however, future work in this area should better tap into the unique barriers to medication adherence among individuals experiencing homelessness, including but not limited to insufficient storage space, loss of medication due to having a transient lifestyle, exchanging medication for other goods, lack of transportation, and other cost-related barriers. Within this sample, insured individuals were more likely to report “other reasons” for not taking their depression medication, suggesting that insurance may lend itself to increased access to alternative methods of treatment beyond psychiatric medication. Future studies may benefit from the use of tailored response items and/or the use of qualitative methods, as these may be more adept in identifying the nuances that underlie reasons for depression and anxiety medication nonadherence. Better understanding these issues may inform intervention development.

Finally, we hypothesized that younger age would be associated with greater odds of medication nonadherence. Results from this study yielded null results; however, despite statistical insignificance, the age of the individuals who endorsed either depression medication or anxiety medication nonadherence appeared to be consistent with the mean age of our sample. Prior research looking at age and medication nonadherence revealed mixed results and thus, findings from our study may support the notion that the association between age and medication nonadherence may not be linear among individuals who are homeless [1,8,14]. It is noteworthy to mention both the depression medication and anxiety medication subgroups were predominately men (58.5% and 57.46%, respectively). However, among this sample of homeless adults, sex did not contribute to a meaningful difference in medication nonadherence across both subgroups. In contrast, results from Tinland and colleagues [14] found that medication nonadherence rates tended to be higher in men, despite the reporting of more subjective side effects by women. Nonetheless, our findings are meaningful in that they highlight the need for interventions and adherence screenings regardless of age or sex.

## 5. Limitations

Several limitations exist for the current study. First, caution should be exercised in generalizing these findings to homeless adults living in other locations, as participants of this study were recruited from homeless serving agencies located in one southern metropolitan city. In addition, data from this study could be considered dated, as they were collected during the summer of 2016. Notwithstanding, results should be replicated with other samples and future studies might consider qualitative methods to further understand barriers related to medication adherence and potential mechanisms to address them among this vulnerable population. Another limitation was the inability to determine a causal relationship due to the cross-sectional nature of the data. Further, as data collection consisted of self-reports, response biases should not be discounted as a limitation. As depression and anxiety medication adherence were each assessed via a single item regarding adherence over the past day, findings may not account for overall medication adherence across time. Future studies may choose to utilize items that account for medication adherence over an extended period of time; however, doing so may lead to introspective recall bias. Other methods for adherence monitoring in the literature include electronic bottle caps, pharmacy data, or pill counts, and can be considered in future studies [30]. Additionally, low endorsements for some racial/ethnic groups (e.g., Hispanic) necessitated collapsing of >1 race/ethnicity in the same category; this is not ideal for reasons including that subgroups within collapsed categories have distinct cultural identities. However, a comparison with the racial/ethnic proportions of the Oklahoma City homeless population as reported in a 2016 point-in-time count demonstrates rough alignment with our subsample composition (race: 58% White, 30% Black, 7% Native American, 5% all other/multi-races, with 1% Asian; ethnicity: 7% Hispanic but collapsed within race) [31]. Future studies would benefit from over-recruitment of race/ethnicities less prevalent among those experiencing homelessness. Relatedly, although the composition of the underlying convenience sample represented ~38.45% of the adult homeless population in Oklahoma City at the time of data collection [31], the extent to which our depression/anxiety sample subsets were reflective of prescription rates amongst the underlying homeless population is unknown. Lastly, a longitudinal study design may be beneficial to understanding medication adherence, as well as the barriers to medication adherence over time.

## 6. Conclusions

In conclusion, and despite the study’s limitations, findings from this study address an important gap within the literature by shedding light on the association between sociodemographic characteristics and depression and anxiety medication nonadherence among a large and diverse sample of homeless adults. Furthermore, this study highlights the need to better understand the unique barriers homeless individuals face with regard to medication adherence, so as to minimize and mitigate sub-optimal health outcomes among this marginalized group.

## Figures and Tables

**Table 1:** Participant Demographics by Depression Medication Adherence Status.

	All	Depression Medication Adherence		*p*-Value
		Yes	No	
	(*n* = 200)	(*n* = 153)	(*n* = 47)	
	Mean (SD)/N [%]			
Age	43.98 (12.08)	43.69 (12.1)	44.93 (12.1)	0.5415
Education (Years)	12.07 (1.95)	12.16 (1.73)	11.79 (2.53)	0.3523
Last Month’s Income (USD)	291.21 (499.01)	232.52 (387.97)	481.98 (728.95)	0.0342
Sex				0.3984
Male	58.50 [117]	60.13 [92]	53.19 [25]	
Female	41.50 [83]	39.87 [61]	46.81 [22]	
Race/Ethnicity				0.0018
White	59.39 [117]	62.91 [95]	47.83 [22]	
Black or African American	16.24 [32]	11.92 [18]	30.43 [14]	
Native American/Alaska Native	8.63 [17]	6.62 [10]	15.22 [7]	
Hispanic/Multi-racial/Other	15.74 [31]	18.54 [28]	6.52 [3]	
Employment				0.0954
Unemployed	89.50 [179]	91.50 [140]	82.98 [39]	
Employed	10.50 [21]	8.50 [13]	17.02 [8]	
Insurance				
Uninsured	70.00 [140]	75.82 [116]	51.06 [24]	0.0012
Insured	30 [60]	24.18 [37]	48.94 [23]	
Depression Anxiety Comorbidity *				
No	27.50 [55]	28.10 [43]	25.53 [12]	
Yes	72.50 [145]	71.90 [110]	74.47 [35]	0.7297

Note. * = based on active psychiatric prescription for each condition. No individuals reporting Asian descent were among this subsample.

**Table 2:** Logistic Regression Predicting Likelihood of Reporting Medication Nonadherence—Depression.

Depression	AOR (95%CI) Ref Group = Adherent	*p*-Value
Age	1.000 (0.966, 1.034)	0.9916
Sex Female (Ref: Male)	1.228 (0.555, 2.718)	0.6119
Black or African American (Ref: Non-Hispanic White)	3.371 (1.246, 9.120)	0.0167
Hispanic/Multi-racial/Other (Ref: Non-Hispanic White)	0.428 (0.110, 1.665)	0.2207
Native American/Alaska Native (Ref: Non-Hispanic White)	3.713 (1.132, 12.186)	0.0305
Education (Years)	1.004 (0.805, 1.253)	0.9689
Last Month’s Income (USD)	1.001 (1.000, 1.001)	0.1667
Employment (Ref: Unemployed)	4.022 (1.244, 13.004)	0.0201
Insurance (Ref: Uninsured)	2.923 (1.225, 6.973)	0.0156
Depression Anxiety Comorbidity * (Ref: No)	1.169 (0.487, 2.805)	0.7266

Note. AOR: adjusted odds ratio; CI: confidence interval. * = based on active psychiatric prescription for each condition. No individuals reporting Asian descent were among this subsample.

**Table 3:** Participant Demographics by Anxiety Medication Adherence Status.

	All	Anxiety Medication Adherence		*p*-Value
		Yes	No	
	(*n* = 181)	(*n* = 121)	(*n* = 60)	
	Mean (SD)/N [%]			
Age	43.45 (11.02)	43.08 (10.88)	44.22 (11.37)	0.5150
Education (Years)	12.06 (1.71)	12.00 (1.54)	12.18 (2.03)	0.5385
Last Month’s Income ($)	348.66 (625.43)	272.88 (542.91)	513.35 (755.11)	0.0424
Sex				0.6263
Male	57.46 [104]	56.20 [68]	60.00 [36]	
Female	42.54 [77]	43.80 [53]	40.00 [24]	
Race/Ethnicity				0.0253
White	54.44 [98]	58.68 [71]	45.76 [27]	
Black or African American	17.22 [31]	14.05 [17]	23.73 [14]	
Native American/Alaska Native	11.11 [20]	7.44 [9]	18.64 [11]	
Hispanic/Multi-racial/Other	17.22 [1]	19.83 [24]	11.86 [7]	
Employment				0.0896
Unemployed	88.95 [161]	91.74 [111]	83.33 [50]	
Employed	11.05 [20]	8.26 [10]	16.67 [10]	
Insurance				0.0235
Uninsured	66.30 [120]	71.90 [87]	55.00 [33]	
Insured	33.70 [61]	28.10 [34]	45.00 [27]	
Depression Anxiety Comorbidity *				0.0003
No	19.89 [36]	12.40 [15]	35.00 [21]	
Yes	80.11 [145]	87.60 [106]	65.00 [39]	

Note. * = based on active psychiatric prescription for each condition. No individuals reporting Asian descent were among this subsample.

**Table 4:** Logistic Regression Predicting Likelihood of Reporting Medication Nonadherence—Anxiety.

Anxiety	AOR (95% CI) Ref Group = Adherent	*p*-Value
Age	1.002 (0.967, 1.039)	0.8934
Sex Female (Ref: Male)	0.832 (0.382, 1.810)	0.6421
Black or African American (Ref: Non-Hispanic White)	1.928 (0.706, 5.264)	0.2004
Hispanic/Multi-racial/Other (Ref: Non-Hispanic White)	0.471 (0.147, 1.514)	0.2065
Native American/Alaska Native (Ref: Non-Hispanic White)	2.980 (0.957, 9.279)	0.0595
Education (Years)	1.131 (0.899, 1.422)	0.2942
Last Month’s Income (USD)	1.000 (1.000, 1.001)	0.4162
Employment (Ref: Unemployed)	3.573 (1.160, 11.010)	0.0266
Insurance (Ref: Uninsured)	2.109 (0.903, 4.927)	0.0846
Depression Anxiety Comorbidity * (Ref: No)	0.333 (0.137, 0.810)	0.0153

Note. AOR: adjusted odds ratio; CI: confidence interval. * = based on active psychiatric prescription for each condition. No individuals reporting Asian descent were among this subsample.

**Table 5:** Frequency of Reasons for Psychiatric Medication Nonadherence.

	Depression	Anxiety
Reason	*n* = 47	*n* = 60
	*n* (%)	
Side effects	2 (4.26)	5 (8.33)
Forgot	7 (14.89)	5 (8.33)
Medication not working	3 (6.38)	2 (3.33)
Doesn’t need the medication anymore	4 (8.51)	9 (15.00)
Haven’t picked up from pharmacy	11 (23.4)	10 (16.67)
Cannot get the medication refilled	11 (23.4)	18 (30.00)
Other reasons	15 (31.91)	22 (36.67)

## Data Availability

The data presented in this study are available on request from the corresponding author. The data are not publicly available due to ethical restrictions based on informed consent agreements.
